# Diazoxide for Neonatal Hyperinsulinemic Hypoglycemia and Pulmonary Hypertension

**DOI:** 10.3390/children10010005

**Published:** 2022-12-21

**Authors:** Shoshana Newman-Lindsay, Satyan Lakshminrusimha, Deepika Sankaran

**Affiliations:** Department of Pediatrics, University of California, Davis, Sacramento, CA 95817, USA

**Keywords:** hyperinsulinism, diazoxide, pulmonary hypertension, hyperinsulinemic hypoglycemia

## Abstract

Hypoglycemia in neonates is associated with long-term neurodevelopmental effects. Hyperinsulinemic hypoglycemia (HH) is the most common cause of persistent hypoglycemia in neonatal intensive care units. Diazoxide is the only medication that is currently recommended for treatment of HH in neonates. However, the use of diazoxide in neonates is associated with pulmonary hypertension as an adverse effect. In this article, we review the literature on the mechanism of action and adverse effects with the use of diazoxide in neonatal hyperinsulinism. We then present a case series of neonates treated with diazoxide in our neonatal intensive care unit over a 5-year period. Among 23 neonates who received diazoxide, 4 developed pulmonary hypertension and 1 died. All infants who developed pulmonary hypertension were born preterm at less than 36 weeks gestation and had pre-existing risk factors for pulmonary hypertension. HH in preterm neonates, with pre-existing pulmonary hypertension or with risk factors for pulmonary hypertension requires thoughtful management.

## 1. Introduction

Prolonged exposure to hypoglycemia is associated with long-term neurocognitive effects in newborns [1,2,3]. Although transient neonatal hypoglycemia improves with intermittent feeds in the newborn (after continuous glucose supply from the placenta into the fetus), some neonates develop persistent hypoglycemia which may be refractory to intravenous dextrose therapy. Diazoxide use in neonatal intensive care units (NICUs) has increased in the past decade for hyperinsulinemic hypoglycemia (HH) [4], the most common cause of persistent hypoglycemia in this setting. In addition to the increase in use of diazoxide, concern about side effects, particularly pulmonary hypertension, has increased [5,6,7]. We present a narrative review describing diazoxide pharmacology, use in HH, association with pulmonary hypertension including possible molecular mechanisms and other reported adverse effects. We follow with a case series from our institution on infants treated with diazoxide in our NICU.

## 2. Pharmacology of Diazoxide

### 2.1. Mechanism of Action

**Figure 1:**
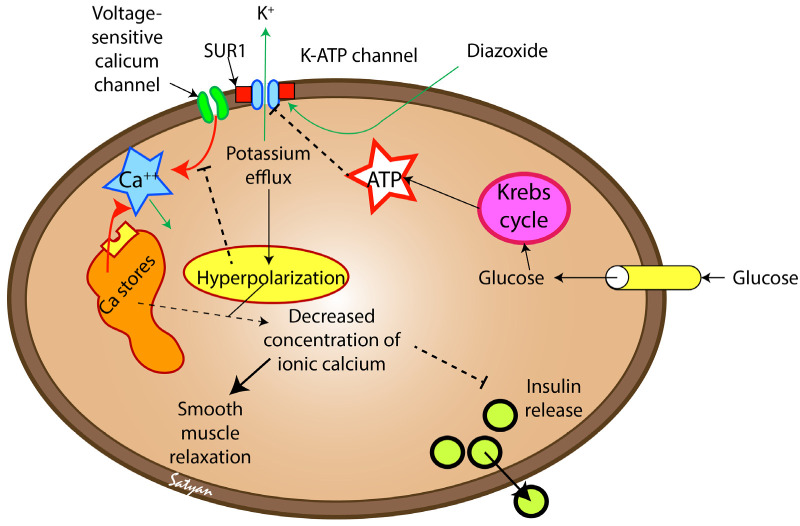
Mechanism of action of diazoxide. Diazoxide stimulates the K-ATP channel, resulting in an efflux of potassium from the beta cells of the pancreas as well as the arterial smooth muscle cells. This efflux results in hyperpolarization of the cell and reduced influx of calcium through the voltage-sensitive calcium channel. Reduced ionic calcium concentration in the cell results in inhibition of insulin release in pancreatic beta cells and smooth muscle relaxation in arterioles. SUR-1 sulfonylurea receptor. Glucose transport into the cell triggers ATP release through the Krebs cycle pathway leading to closure of K-ATP channels resulting in depolarization of the cell, calcium influx and insulin release. Copyright Satyan Lakshminrusimha.

Diazoxide is a nondiuretic benzothiazidinic systemic vasodilator originally formulated for severe hypertension [8,9]. Diazoxide causes smooth muscle relaxation leading to systemic and pulmonary vasodilation [9]. Diazoxide was noted to be diabetogenic in the 1960s, and it was suspected that diazoxide blocks insulin release [10]. Diazoxide raises glucose concentrations by suppressing insulin secretion (Figure 1). The mechanism of action of diazoxide is by blocking sulfonylurea receptor 1 subunit of K_ATP_-channel on pancreatic beta (ẞ) cells, which increases the permeability to potassium ions, resulting in hyperpolarization of the cells. Hyperpolarization of the ẞ-cells inhibits calcium-dependent insulin secretion [6,9]. These K_ATP_-channels are also involved in smooth muscle relaxation and dilation of coronary arterioles [11], so diazoxide causes arteriolar dilation with little to no effect on the venous system [12].

### 2.2. Pharmacokinetics and Dosing of Diazoxide 

Pharmacokinetics of diazoxide was described via a one-compartment model, in which volume of distribution and clearance are proportional to body weight. The plasma half-life in adults is 21–35 h. Diazoxide is highly bound to plasma proteins in adults but is less bound to plasma proteins in neonates, resulting in shorter plasma half-life [13]. Clearance is faster in females [11,12]. Diazoxide takes 72–96 h to reach steady state. Two or three daily doses are equally effective [12]. Diazoxide is metabolized by the oxidation of 3-methyl to hydroxymethyl and carboxy derivatives followed by sulfate conjugation of the hydroxy methyl, and subsequent excretion in urine.

The use of diazoxide to treat hyperinsulinism was first described in 1964. Twice-daily 8 mg/kg/day doses ameliorated hypoglycemia in a 4-year-old child presenting with seizure and refractory hypoglycemia, who was later diagnosed with leucine-sensitivity [14]. A recent series of 20 infants treated with diazoxide in Arkansas showed dosages of 10 ± 3.7 mg/kg/d and a typical duration of 44.9 ± 27.9 days [15], consistent with the Pediatric Endocrinology Society (PES) consensus guidelines published in 2020, in which the recommended dose range for diazoxide is 5–15 mg/kg/d in 2 to 3 divided doses. The starting dose should be selected according to the suspected cause. Lower doses are preferred for neonates with suspected perinatal stress- (or asphyxia-) induced HH and/or underlying cardiac disease [16]. Dosages recommended in Japan are slightly lower (5–10 mg/kg/d before 1 year of age and 3–5 mg/kg/d thereafter) [17]. Doses greater than 15 mg/kg/d are associated with an increased risk of complications [8].

## 3. Diazoxide Use in Hyperinsulinism

### 3.1. Hyperinsulinemic Hypoglycemia 

Hypoglycemia in newborn is associated with long-term brain injury [1,3,18]. Persistent hypoglycemia in neonates is often due to HH associated with birth asphyxia, fetal growth restriction (FGR) or pre-eclampsia [2]. Infants with FGR may have a combination of failure of counter-regulation, immaturity of the enzyme systems regulating gluconeogenesis, glycogenolysis, ketogenesis, reduced adipocyte stores and hyperinsulinism [19]. If not identified and treated in a timely manner, HH may affect cognition and neurodevelopment due to deprivation of glucose as the critical brain fuel. Moreover, the inhibitory effect of insulin on lipolysis and ketogenesis prevents the formation of alternative fuel sources for the brain. Although HH can be a transient condition, it can persist for several months [20]. The detection of insulin in plasma in a hypoglycemic infant is characteristic of HH.

In an emergent setting, parenteral dextrose infusion is used for management of hypoglycemia. A dextrose bolus may be necessary initially, but repeated boluses may potentially trigger more insulin secretion. Delivery of glucose as a continuous intravenous infusion starting at 4–8 mg/kg/min is currently the standard in most NICUs, although patients with HH may require greater than 25 mg/kg/min to maintain normoglycemia [21]. Glucagon, given as 0.5–1 mg via intramuscular or subcutaneous injection, is a first-line therapy if neonates are unable to tolerate oral feeds or intravenous access is difficulty to obtain [21]. Frequent feeding, extended feeds over 60–150 min, and continuous gavage feeds have also been used to improve glucose stability [21].

### 3.2. Diazoxide in Hyperinsulinemia Hypoglycemia 

Diazoxide is the only agent approved by the FDA for treatment of hypoglycemia due to hyperinsulinism. Although the approval is for a specific subset of conditions (leucine sensitivity, islet cell hyperplasia, extrahepatic malignancy, islet cell adenoma and adenomatosis) [22], it is often used as a first-line treatment for all types of neonatal HH including transient HH [4,20,23]. Octreotide is an additional agent that is used either as a continuous infusion or 3–4 subcutaneous doses per day for HH. Lanreotide, a long-acting somatostatin analog, reduces insulin gene promoter activity to decrease insulin production [21] in neonates with HH.

Treatment of HH with diazoxide is recommended by pediatric endocrinologists to prevent long-term neurodevelopmental consequences of persistent hypoglycemia. For growth-restricted infants, Pediatric Endocrinology Society guidelines recommend delaying the consideration of diazoxide until 7–10 days of life since the HH in IUGR infants may take weeks to normalize. Concurrent use of a thiazide diuretic to minimize pulmonary edema is also recommended [16]. Diazoxide responsive patients demonstrate a significant increase in serum glucose and end their dependence on intravenous glucose [9] or achieve the ability to maintain glucose concentrations greater than 70 mg/dL (3.9 mmol/L) during an age-appropriate fast, or during ketonemia (>2 mmol/L) (associated with plasma glucose concentrations greater than 50 mg/dL (2 mmol/L) [24].

A recent systematic review that screened 161 articles, and ultimately included the only clinical trial conducted in India in which 30 low-birthweight infants with HH within 5 days after birth were randomized to either oral diazoxide (9 mg/kg/day in 3 divided doses, increased to 12 mg/kg/day if hypoglycemia persisted after 48 h) or placebo [25,26]. Low certainty of evidence suggested that infants treated with diazoxide had a shorter duration of intravenous dextrose therapy (114 ± 51 hours with diazoxide vs. 164 ± 71hours with placebo, 95% CI[−94,−6]) and shorter time to achieve full enteral feeds (117 ± 51 with diazoxide vs. 166 ± 65 hours with placebo, 95% CI[−91, −7])[26]. There were no data available from this systematic review for episodes of critical hypo- or hyperglycemia after initiation of diazoxide, length of hospitalization, pulmonary hypertension or congestive heart failure, and neurodevelopmental outcomes.

Diazoxide-responsiveness varies with underlying etiology of HH. In a large cohort from Japan, over 96% of infants with transient HH had a good response to diazoxide with improvements in hypoglycemia compared with 57% of infants with permanent or genetic causes of HH [17]. A retrospective study of 141 neonates with transient HH including 34 (24%) neonates treated with diazoxide concluded that higher plasma C-peptide concentrations (>1.4 ± 0.9 ng/mL) on the “critical blood sample” obtained at the time of hypoglycemia and higher maximal glucose infusion rate (GIR > 16.6 ± 3.4 mg/kg/min) requirement may serve as clinical tools to predict diazoxide responsiveness among neonates with transient HH [27]. Diazoxide is effective in transient HH, and useful for permanent and genetic etiologies of diffuse HH in which the K_ATP_ channel function is intact [28]. In contrast, diazoxide is not effective in HH due to recessive (and a few dominant) inactivating mutations in *ABCC8* and *KCNJ11* genes and in patients with focal HH (paternally inherited *ABCC8* or *KCNJ11* mutation and a paternal isodisomy of the 11p15 region, which is specific to the islets cells within the focal lesion) [29]. Focal lesions are diagnosed using genetic testing and ^18^F-fluoro-L-DOPA positron emission tomography (PET scan). Hypoglycemia must be rapidly and intensively managed to prevent severe and irreversible brain damage using intravenous dextrose solution with high GIR and intramuscular glucagon. Surgical treatment with partial pancreatectomy is indicated in focal HH.

### 3.3. Diazoxide Use in HH and Cardiovascular Complications 

Yoshida et al. reported circulatory complications (edema, oliguria and reopening of ductus arteriosus) in 10/64 neonates with transient HH and 5/14 neonates with permanent HH treated with diazoxide in a questionnaire-based study conducted among Japanese neonatologists in a retrospective design [17]. In this study, younger gestational age (i.e., prematurity) and higher maximum dose of diazoxide were significant risk factors for circulatory complications. The authors suggested that prophylactic use of diuretics may prevent cardiovascular complications after diazoxide therapy in very-low-birthweight infants and in neonates with congenital heart disease [30]. This supports the recommendation to provide concurrent thiazide diuretics to minimize pulmonary edema. In another retrospective analysis of 194 neonates with HH among whom 165 (85.1%) received diazoxide therapy, 17 neonates had serious adverse effects and 8/17 had pulmonary hypertension [31]. Moreover, the rate of serious adverse effects was higher among perinatal stress-induced HH compared with otherwise healthy newborns with genetic causes of HH.

In 2015, the United States Food and Drug Administration (FDA) issued a black box warning about the association between diazoxide use and pulmonary hypertension [32]. This was based on 11 cases identified by the FDA where diazoxide use was complicated by pulmonary hypertension [33,34,35]. Recent studies report incidence of pulmonary hypertension in 2–7% of pediatric patients with HH treated with diazoxide [6,7]. However, case series and cohort studies of neonates suggest that in certain infants with pre-existing risk factors, the rate of pulmonary hypertension following diazoxide therapy may be higher [8,36] Case series have also reported reopening of the ductus arteriosus [17] in diazoxide-exposed neonates. Prematurity, structural heart disease and pre-existing pulmonary hypertension have been identified as potential risk factors for diazoxide-associated exacerbation of pulmonary hypertension [6,7].

### 3.4. Diazoxide and Necrotizing Enterocolitis

Diazoxide use has been associated with an increased risk of necrotizing enterocolitis (NEC) in two small cohorts [5,37] and a case report [38]. Keyes et al. reported on 24 diazoxide-exposed patients in the Massachusetts General Hospital NICU, of whom 5 developed NEC. This 20% NEC rate was significantly higher than the baseline incidence of 1% overall and 6% among inborn very-low-birthweight infants in their NICU [5]. Prado et al. reported similar findings among three tertiary NICUs in Toronto, Canada: 55 patients were treated with diazoxide and 7 (13%) developed NEC, with diazoxide exposure being associated with increased odds of NEC compared with non-exposed infants of a similar gestational age [37]. Larger cohorts have reported much lower rates of NEC among diazoxide exposed infants [2,4,7]; however, these lacked information on baseline NEC rates or included diverse populations beyond the neonatal period.

## 4. Pathogenesis of Pulmonary Hypertension and Heart Failure after Diazoxide Use

The pathogenesis of pulmonary hypertension following diazoxide use is not well-elucidated. Diazoxide was initially investigated as a treatment for pulmonary hypertension when a case report described a promising response in a young woman with idiopathic pulmonary hypertension. The direct injection of diazoxide into the pulmonary artery during cardiac catheterization decreased pulmonary artery resistance and increased cardiac output. The patient was transitioned to oral diazoxide and had a good outcome 6 months post-procedure [39]. However, a subsequent case-series showed it to be ineffective in treatment of pulmonary hypertension along with occurrence of significant adverse effects [40].

**Figure 2:**
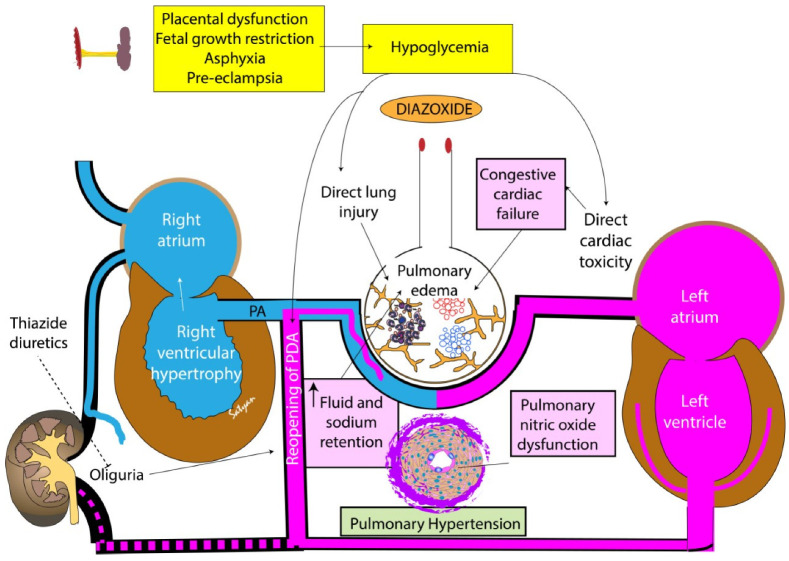
Potential mechanisms of pulmonary hypertension following diazoxide use in neonates. Neonates with hypoglycemia have antecedent risk factors such as placental dysfunction, fetal growth restriction (FGR), pre-eclampsia and asphyxia that are also risk factors for pulmonary hypertension. Diazoxide causes oliguria, fluid and sodium retention leading to congestive cardiac failure and pulmonary edema. Reopening of patent ductus arteriosus (PDA) can also contribute to hyperdynamic pulmonary hypertension and pulmonary edema. Abnormalities in pulmonary vascular nitric oxide pathway have been speculated to be secondary to diazoxide and contribute to pulmonary hypertension. Treatment with thiazide diuretics may ameliorate or prevent diazoxide-induced pulmonary hypertension. PA—pulmonary artery. Copyright Satyan Lakshminrusimha.

Diazoxide affects several types of K_ATP_ channels, including those in the ventricles in the heart, pancreatic beta cells and in mitochondria [11]. The pathogenesis of pulmonary hypertension may potentially be related to direct cardiac and pulmonary toxicity (Figure 2) [8]. Diazoxide has been shown to significantly decrease inducible nitric oxide synthase (iNOS) activity by increasing its phosphorylation in skeletal muscles affected by ischemia-reperfusion injury [41]. We speculate that diazoxide may decrease iNOS activity in pulmonary arterial smooth muscle cells similar to the above-described action on skeletal muscle cells, leading to reduced endogenous nitric oxide generation resulting in pulmonary hypertension. Pathological evidence of pulmonary vascular toxicity on lung biopsy was reported by Nebesio et al. [42]. Diazoxide therapy can cause sodium retention and decrease free water clearance, leading to fluid retention. The congestive heart failure associated with diazoxide use occurs in the setting of fluid retention and volume overload [8,43]. This is seen less frequently with concurrent administration of a thiazide diuretic to counteract salt and water retention. Although the mechanism is not understood, one study showed an association between higher total fluid volume in the 24 h preceding diazoxide administration (150 mL/kg/d vs. 130 mL/kg/d) and increased risk of pulmonary hypertension [6]. Pulmonary hypertension and congestive heart failure can occur simultaneously following diazoxide therapy [34,35] and improve upon discontinuation of diazoxide therapy. Simultaneous use of diuretics (such as hydrochlorothiazide) has been suggested to avoid fluid retention in neonates on diazoxide therapy.

## 5. Other Adverse Effects of Diazoxide Use in Neonates

Adverse effects of diazoxide use in neonates include edema, hyperuricemia, tachycardia, hypertrichosis, leukopenia, feeding intolerance as well as the cardiovascular effects discussed above. The sodium and fluid imbalances caused by diazoxide are likely due to action on vasculature rather than direct renal effects, as direct injection of diazoxide into the renal artery of rats increased natriuresis [44]. Consensus guidelines recommend monitoring for pulmonary hypertension with an echocardiogram about a week after starting diazoxide, monitoring for neutropenia and thrombocytopenia (observed in about 15% infants treated with diazoxide) with a complete blood count at baseline, five to seven days after initiation and every three to six months thereafter. Due to the risk of hyperuricemia, uric acid levels should also be checked at baseline, after five to seven days and then every six months [16].

Plasma diazoxide concentrations were not associated with differences in rate of adverse effects in pediatric age group in a prospective multicenter study conducted in Japan. However, high diazoxide concentrations greater than 100 mcg/mL were associated with hyperglycemia [12]. Other reported adverse effects of diazoxide include vomiting, dependent edema, tachycardia, neutropenia, cardiopulmonary failure and rarely seizures. Diazoxide is >90% bound to plasma proteins; therefore, hypoalbuminemia, especially in the setting of liver failure, can increase free or unbound diazoxide concentrations, increasing the risk of adverse effects [45]. A case report described a 35-week gestation infant with perinatal metabolic acidosis and HH who developed cardiorespiratory failure with pulmonary hypertension, hepatomegaly, macroglossia and hypertrichosis following the initiation of diazoxide, in the setting of liver failure with hypoalbuminemia [45]. The symptoms quickly resolved within 72 h after discontinuation of diazoxide. Moreover, renal failure may also predispose to diazoxide toxicity due to decreased renal excretion of the drug. On the contrary, diazoxide use was associated with acute kidney injury with elevated serum creatinine in a neonate, without affecting urine output in a case report [46]. Pericardial effusions have been reported in association with diazoxide exposure in a small number of patients [47,48], including a 7-week-old who required diuretics and an 8-month-old who required emergency subxiphoid drainage. Diazoxide was discontinued in both patients and the effusions did not recur [47].

## 6. Case Series from a Single Center (University of California Davis NICU)

Newborn infants treated with diazoxide between July 2017 and July 2022 in the NICU at University of California, Davis were retrospectively identified from the electronic medical record. Data on gestational age and weight at birth, timing of initiation of diazoxide and its dosage and, as well as clinical course including any change in respiratory support and risk factors for pulmonary hypertension, were extracted from the electronic medical record. In our study, pulmonary hypertension was defined as need for increased respiratory support coupled with characteristic echocardiographic changes (increased tricuspid regurgitant jet velocity and/or increased interventricular septal flattening with elevated right ventricular systolic pressures). The institutional review board at University of California, Davis, deemed this study exempt from review.

Twenty-three neonates were treated with diazoxide over a five-year period. Twenty of these twenty-three had non-genetic caused of HH, and all 20 neonates had improved blood glucose stability on diazoxide (Table 1). Of these 23 neonates, 4 (17%) developed pulmonary hypertension associated with diazoxide (supplementary table) and one infant died due to a pulmonary hypertensive crisis, despite extracorporeal membrane oxygenation (ECMO) (Table 1). Two additional preterm neonates exhibited echocardiographic changes in pulmonary hypertension and required increased respiratory support after diazoxide treatment not attributed to the medication. One infant had congenital pulmonary airway malformation and the other infant had sepsis and necrotizing enterocolitis. Neonates who developed pulmonary hypertension showed echocardiographic changes after diazoxide administration that preceded the need for prolonged respiratory support. Our cohort included 12 neonates born at less than 36 completed weeks, and all cases of pulmonary hypertension occurred among these infants. One full term neonate showed echocardiographic changes of pulmonary hypertension without need for increased respiratory support.

Of the 23 neonates, 5 (17% of all neonates) and 4/12 (33% of less than 36-week gestation neonates) treated with diazoxide for HH developed pulmonary hypertension requiring increased respiratory support and prolonged NICU stay (Table S1). None of the term infants had complications from diazoxide. This rate is much higher than the 2–3% reported in larger heterogenous populations and similar to the 5/15 neonates who developed pulmonary hypertension with diazoxide described by Desai et al. [36]. All four of these infants had pre-existing risk factors for pulmonary hypertension, including premature birth in 4/4 and prior diagnosis of pulmonary hypertension in 2/4.

## 7. Current Evidence on Diazoxide Use in Neonates

Diazoxide directly targets dysregulated insulin secretion (which is the primary etiology for neonatal HH) by the activation of mitochondrial ATP-sensitive potassium channels on pancreatic ẞ cells resulting in attenuated glucose-stimulated insulin release. Furthermore, it has good oral bioavailability and is inexpensive. Diazoxide is the medication of choice in genetic causes of HH with intact K_ATP_ channels and for transient refractory HH that does not improve with intravenous dextrose infusion at high GIR and extended or continuous feeds. However, the safety profile of diazoxide in neonatal HH is not established. Due to the risk of cardiorespiratory complications, all infants treated with diazoxide should be monitored with screening echocardiography prior to initiating and 1 week after initiation of treatment according to PES guidelines; diazoxide should be avoided if pulmonary hypertension is noted on baseline echocardiogram. However, echocardiographic changes of pulmonary hypertension preceded the need for increased respiratory support in our series of neonates with HH. We suggest that diazoxide treatment should be discontinued if the echocardiogram demonstrates new-onset or worsening pulmonary hypertension.

In order to decrease the risk of pulmonary hypertension as an adverse effect of diazoxide, it has been proposed to add chlorothiazide to prevent fluid retention. Additionally, avoiding the use of diazoxide infants with pre-existing pulmonary hypertension and liver injury may be beneficial in preventing cardiovascular complications from diazoxide toxicity [45]. Meconium aspiration syndrome, pneumonia and sepsis may be associated with pulmonary hypertension as a complication; therefore, it would be reasonable to be cautious with diazoxide and prioritize supportive care or other medications for hypoglycemia in these patients. Randomized clinical trials that are adequately powered to assess for cardiorespiratory complications of diazoxide in HH are warranted.

Potential areas for future research include identifying appropriate management strategies for infants with risk factors for pulmonary hypertension who remain hypoglycemic despite intravenous dextrose and/or continuous feeds, as well as investigating ways to promote optimal neurodevelopmental outcomes for sick and hypoglycemic infants. Neuroprotective effects of diazoxide have been demonstrated in animal models and need further investigation both in asphyxiated term neonates and in preterm infants. In a rat model, diazoxide promoted the proliferation and myelination of oligodendrocyte precursor cells and may prevent hypoxia-induced periventricular white matter injury [49]. In a newborn piglet model, diazoxide protects postischemic vascular reactivity to carbon dioxide, an indicator of endothelial function in ischemia–reperfusion injury, contributing to neuroprotection [50]. Additionally, diazoxide preserves neuronal function by enhanced cellular protection after ischemia/reperfusion by activation of mitochondrial K_ATP_ channels and preconditioning of cerebral endothelium and a protects the blood–brain barrier from ischemic stress [51,52], potentially via the PI3K/AKT pathway [53].

## 8. Conclusions

Experience from our neonatal unit adds to the growing concern on the safety profile of diazoxide. We found that echocardiographic evidence of pulmonary hypertension precedes an increase in respiratory support in neonates treated with diazoxide. Risk factors for hypoglycemia are often associated with pulmonary hypertension (Figure 2). Obtaining an echocardiogram prior to diazoxide therapy and 1 week after the initiation of diazoxide therapy may be helpful in the early identification of pulmonary hypertension. Additionally, avoiding the use of higher doses of diazoxide, taking care to delay initiation until after the first week of life, or choosing alternate therapeutic modalities in specific subpopulations of newborn infants who are predisposed to develop pulmonary hypertension (such as preterm infants, asphyxiated infants treated with therapeutic hypothermia, neonates with structural heart disease and those with pre-existing pulmonary hypertension) may be beneficial and needs further investigation.

## Figures and Tables

**Table 1:** Characteristics of neonates who did and did not develop pulmonary hypertension after Diazoxide.

Characteristic	Pulmonary Hypertension (*n* = 4)	No Pulmonary Hypertension (*n* = 17)	*p*-Value
Type of HH Transient Permanent	4 (100%) 0	16 (94%) 1 (6%)	
Gestational Age (weeks) Mean (SD)	31.25 (3.9)	35.29 (3.3)	*p* = 0.043
Birthweight (g) Mean (SD)	1320 (911)	1965 (672)	*p* = 0.12
IUGR Yes Yes, with AEDF/REDF *	3 (75%) 2 (50%)	9 (53%) 1 (6%)	*p* = 0.6
Age at Diazoxide Start <7 days 7–14 days >14 days	1/4 (25%) 1/4 (25%) 2/4 (50%)	5/17 (30%) 5/17 (30%) 7/17 (40%)	*p* = 0.9
Starting Diazoxide Dose 10 mg/kg/d 5–9 mg/kg/d Less than 5 mg/kg/d	3 (75%) 1 (25%) 0	4 (24%) 12 (70%) 1 (6%)	*p* = 0.08
Prior Echocardiography Echo prior to diazoxide Mild Septal Flattening TR jet estimate > 45 mmHg	4 (100%) 2 (50%) 1 (25%)	11 (65%) 1 (6%) 1 ** (6%)	*p* = 0.07
Respiratory Support at Diazoxide Start Room Air Respiratory Support Nasal Cannula CPAP Invasive Mechanical Ventilation	1 (25%) 3 (75%) 1 2 0	16 (94% 1 (6%) 0 0 1 **	*p* = 0.01

Twenty-one of the twenty-three exposed neonates are presented in this table. Two infants who were exposed to diazoxide and subsequently developed pulmonary hypertension that was not attributed to diazoxide by the treating neonatal care team, were excluded from this analysis. The *p*-values were calculated via *t*-test for continuous variables and Fisher’s exact test for categorical variables. * Absent/Reversed End Diastolic Flow. ** One patient, acutely ill with congenital syphilis, who was on high-frequency ventilation and inhaled nitric oxide and received one dose of diazoxide for hyperinsulinemic hypoglycemia. This patient showed rapid improvement as infection was treated. IUGR, intrauterine growth restriction. Echo, echocardiogram. TR, tricuspid regurgitation. CPAP, continuous positive airway pressure.

## Data Availability

The data presented in this study are available in this article.

## Supplementary Materials

The following supporting information can be downloaded at: https://www.mdpi.com/article/10.3390/children10010005/s1, Table S1: Clinical and echocardiographic characteristics of neonates who developed pulmonary hypertension following diazoxide therapy.

## References

[1] Güemes M., Hussain K. (2015). Hyperinsulinemic hypoglycemia. Pediatr. Clin..

[2] Thornton P.S., Stanley C.A., De Leon D.D., Harris D., Haymond M.W., Hussain K., Levitsky L.L., Murad M.H., Rozance P.J., Simmons R.A. (2015). Recommendations from the Pediatric Endocrine Society for evaluation and management of persistent hypoglycemia in neonates, infants, and children. J. Pediatr..

[3] Adamkin D.H. (2011). Committee on Fetus and Newborn. Postnatal glucose homeostasis in late-preterm and term infants. Pediatrics.

[4] Gray K.D., Dudash K., Escobar C., Freel C., Harrison T., McMillan C., Puia-Dumitrescu M., Cotten C.M., Benjamin R., Clark R.H. (2018). Prevalence and safety of diazoxide in the neonatal intensive care unit. J. Perinatol..

[5] Keyes M.L., Healy H., Sparger K.A., Orth L.E., Geha M., Roumiantsev S., Matute J.D. (2021). Necrotizing Enterocolitis in Neonates With Hyperinsulinemic Hypoglycemia Treated With Diazoxide. Pediatrics.

[6] Chen S.C., Dastamani A., Pintus D., Yau D., Aftab S., Bath L., Swinburne C., Hunter L., Giardini A., Christov G. (2019). Diazoxide-induced pulmonary hypertension in hyperinsulinaemic hypoglycaemia: Recommendations from a multicentre study in the United Kingdom. Clin. Endocrinol..

[7] Herrera A., Vajravelu M.E., Givler S., Mitteer L., Avitabile C.M., Lord K., De León D.D. (2018). Prevalence of Adverse Events in Children With Congenital Hyperinsulinism Treated With Diazoxide. J. Clin. Endocrinol. Metab..

[8] Timlin M.R., Black A.B., Delaney H.M., Matos R.I., Percival C.S. (2017). Development of Pulmonary Hypertension During Treatment with Diazoxide: A Case Series and Literature Review. Pediatr. Cardiol..

[9] De Cosio A.P., Thornton P. (2019). Current and Emerging Agents for the Treatment of Hypoglycemia in Patients with Congenital Hyperinsulinism. Paediatr. Drugs.

[10] Seltzer H.S., Allen E.W. (1969). Hyperglycemia and inhibition of insulin secretion during administration of diazoxide and trichlormethiazide in man. Diabetes.

[11] Coetzee W.A. (2013). Multiplicity of effectors of the cardioprotective agent, diazoxide. Pharmacol. Ther..

[12] Kizu R., Nishimura K., Sato R., Kosaki K., Tanaka T., Tanigawara Y., Hasegawa T. (2017). Population Pharmacokinetics of Diazoxide in Children with Hyperinsulinemic Hypoglycemia. Horm. Res. Paediatr..

[13] Pruitt A.W., Faraj B.A., Dayton P.G. (1974). Metabolism of diazoxide in man and experimental animals. J. Pharmacol. Exp. Ther..

[14] Drash A., Wolff F. (1964). Drug Therapy in Leucine-Sensitive Hypoglycemia. Metabolism.

[15] Raisingani M., Brar P.C. (2019). Characterization of the duration of treatment with diazoxide in infants with prolonged hyperinsulinism (PHI). J. Pediatr. Endocrinol. Metab..

[16] Brar P.C., Heksch R., Cossen K., De Leon D.D., Kamboj M.K., Marks S.D., Marshall B.A., Miller R., Page L., Stanley T. (2020). Management and Appropriate Use of Diazoxide in Infants and Children with Hyperinsulinism. J.Clin. Endocrinol. Metab..

[17] Yoshida K., Kawai M., Marumo C., Kanazawa H., Matsukura T., Kusuda S., Yorifuji T., Heike T. (2014). High prevalence of severe circulatory complications with diazoxide in premature infants. Neonatology.

[18] Menni F., de Lonlay P., Sevin C., Touati G., Peigné C., Barbier V., Nihoul-Fékété C., Saudubray J.M., Robert J.J. (2001). Neurologic outcomes of 90 neonates and infants with persistent hyperinsulinemic hypoglycemia. Pediatrics.

[19] Fafoula O., Alkhayyat H., Hussain K. (2006). Prolonged hyperinsulinaemic hypoglycaemia in newborns with intrauterine growth retardation. Arch. Dis. Child. Fetal Neonatal Ed..

[20] Hoe F.M., Thornton P.S., Wanner L.A., Steinkrauss L., Simmons R.A., Stanley C.A. (2006). Clinical features and insulin regulation in infants with a syndrome of prolonged neonatal hyperinsulinism. J. Pediatr..

[21] Demirbilek H., Hussain K. (2017). Congenital hyperinsulinism: Diagnosis and treatment update. J. Clin. Res. Pediatr. Endocrinol..

[22] Med D. Label: Proglycem—Diazoxide Suspension. Drug Label Information 2022. https://dailymed.nlm.nih.gov/dailymed/drugInfo.cfm?setid=b16c7832-2fd9-49af-b923-1dc0d91fd6e2.

[23] Hu S., Xu Z., Yan J., Liu M., Sun B., Li W., Sang Y. (2012). The treatment effect of diazoxide on 44 patients with congenital hyperinsulinism. J. Pediatr. Endocrinol. Metab..

[24] Palladino A.A., Stanley C.A. (2011). A specialized team approach to diagnosis and medical versus surgical treatment of infants with congenital hyperinsulinism. Semin. Pediatr. Surg..

[25] Laing D., Hanning S.M., Harding J.E., Mravicich L.C., McKinlay C.J. (2021). Diazoxide for the Treatment of Transitional Neonatal Hypoglycemia: A Systematic Review. J. Neonatol..

[26] Balachandran B., Mukhopadhyay K., Sachdeva N., Walia R., Attri S.V. (2018). Randomised controlled trial of diazoxide for small for gestational age neonates with hyperinsulinaemic hypoglycaemia provided early hypoglycaemic control without adverse effects. Acta Paediatr..

[27] Davidov A.S., Elkon-Tamir E., Haham A., Shefer G., Weintrob N., Oren A., Lebenthal Y., Mandel D., Eyal O. (2020). Higher C-peptide levels and glucose requirements may identify neonates with transient hyperinsulinism hypoglycemia who will benefit from diazoxide treatment. Eur. J. Pediatr..

[28] Aynsley-Green A., Hussain K., Hall J., Saudubray J.M., Nihoul-Fékété C., De Lonlay-Debeney P., Brunelle F., Otonkoski T., Thornton P., Lindley K.J. (2000). Practical management of hyperinsulinism in infancy. Arch. Dis. Child. Fetal Neonatal. Ed..

[29] Arnoux J.B., Verkarre V., Saint-Martin C., Montravers F., Brassier A., Valayannopoulos V., Brunelle F., Fournet J.C., Robert J.J., Aigrain Y. (2011). Congenital hyperinsulinism: Current trends in diagnosis and therapy. Orphanet. J. Rare Dis..

[30] Kapoor R.R., Flanagan S.E., James C., Shield J., Ellard S., Hussain K. (2009). Hyperinsulinaemic hypoglycaemia. Arch. Dis. Child..

[31] Thornton P., Truong L., Reynolds C., Hamby T., Nedrelow J. (2019). Rate of Serious Adverse Events Associated with Diazoxide Treatment of Patients with Hyperinsulinism. Horm. Res. Paediatr..

[32] FDA FDA Drug Safety Communication. https://www.fda.gov/drugs/drug-safety-and-availability/fda-drug-safety-communication-fda-warns-about-serious-lung-condition-infants-and-newborns-treated.

[33] Gerardin M., Denizot S., Texier R., Jolliet P. (2010). Pulmonary Hypertension in new-borns treated with diazoxide: About two cases. Fundamental & Clinical Pharmacology.

[34] Demirel F., Unal S., Çetin I.I., Esen I., Araslı A. (2011). Pulmonary hypertension and reopening of the ductus arteriosus in an infant treated with diazoxide. J. Pediatr. Endocrinol. Metab..

[35] Yildizdas D., Erdem S., Küçükosmanoglu O., Yilmaz M., Yüksel B. (2008). Pulmonary hypertension, heart failure and neutropenia due to diazoxide therapy. Adv. Ther..

[36] Desai J., Key L., Swindall A., Gaston K., Talati A.J. (2021). The danger of diazoxide in the neonatal intensive care unit. Ther. Adv. Drug Saf..

[37] Prado L.A., Castro M., Weisz D.E., Jain A., Belik J. (2021). Necrotising enterocolitis in newborns receiving diazoxide. Arch. Dis. Child. Fetal Neonatal. Ed..

[38] Theodorou C.M., Hirose S. (2020). Necrotizing enterocolitis following diazoxide therapy for persistent neonatal hypoglycemia. J. Pediatr. Surg. Case Rep..

[39] Klinke W.P., Gilbert J.A. (1980). Diazoxide in primary pulmonary hypertension. N. Engl. J. Med..

[40] Buch J., Wennevold A. (1981). Hazards of diazoxide in pulmonary hypertension. Br. Heart J..

[41] Farahini H., Ajami M., Mirzay Razaz J., Azad N., Soleimani M., Ayatollahi S.A., Abotaleb N., Peyrovi H., Pazoki-Toroudi H. (2012). Nitric Oxide is Necessary for Diazoxide Protection Against Ischemic Injury in Skeletal Muscle. Iran. J. Pharm. Res..

[42] Nebesio T.D., Hoover W.C., Caldwell R.L., Nitu M.E., Eugster E.A. (2007). Development of pulmonary hypertension in an infant treated with diazoxide. J. Pediatr. Endocrinol. Metab..

[43] Silvani P., Camporesi A., Mandelli A., Wolfler A., Salvo I. (2004). A case of severe diazoxide toxicity. Paediatr. Anaesth..

[44] Fine L.G., Weber H. (1975). Effect of diazoxide on renal handling of sodium in the rat. Clin. Sci. Mol. Med..

[45] Tas E., Mahmood B., Garibaldi L., Sperling M. (2015). Liver injury may increase the risk of diazoxide toxicity: A case report. Eur. J. Pediatr..

[46] Godinho F., Lewin R., Park J., Losa I. (2015). Case of raised creatinine in a newborn with congenital hyperinsulinism: Diazoxide induced acute kidney injury. Endocrine Abstracts.

[47] Hastings L.A., Preddy J., McCready M., Neville K., Verge C.F. (2020). Pericardial Effusion Associated with Diazoxide Treatment for Congenital Hyperinsulinism. Horm. Res. Paediatr..

[48] Maffre I., Vincenti M., Dalla Vale F., Amouroux C., Werner O., Meilhac A., de Barry G., Amedro P. (2019). Diazoxide Causality Assessment of a Pericardial Effusion in a Child with Kabuki Syndrome. J. Clin. Res. Pediatr. Endocrinol..

[49] Fogal B., McClaskey C., Yan S., Yan H., Rivkees S.A. (2010). Diazoxide promotes oligodendrocyte precursor cell proliferation and myelination. PLoS ONE.

[50] Domoki F., Kis B., Nagy K., Farkas E., Busija D.W., Bari F. (2005). Diazoxide preserves hypercapnia-induced arteriolar vasodilation after global cerebral ischemia in piglets. Am. J. Physiol. Heart Circ. Physiol..

[51] Katakam P.V., Dutta S., Sure V.N., Grovenburg S.M., Gordon A.O., Peterson N.R., Rutkai I., Busija D.W. (2016). Depolarization of mitochondria in neurons promotes activation of nitric oxide synthase and generation of nitric oxide. Am. J. Physiol. Heart Circ. Physiol..

[52] Lenzsér G., Kis B., Bari F., Busija D.W. (2005). Diazoxide preconditioning attenuates global cerebral ischemia-induced blood-brain barrier permeability. Brain Res..

[53] Chen Y., Zeng H., Liu H. (2022). MiR-21 participates in the neuroprotection of diazoxide against hypoxic-ischemia encephalopathy by targeting PDCD4. Brain Inj..

