# Supplementary material for: Diazoxide for Neonatal Hyperinsulinemic Hypoglycemia and Pulmonary Hypertension
Source: Children (Basel). 2022 Dec 21;10(1):5. doi: 10.3390/children10010005 (PMC9856357; doi:10.3390/children10010005)
Supplement: Supplementary file 1 [file children-10-00005-s001.zip › children-2101840-supplementary.pdf]

Table S1: Clinical and echocardiographic characteristics of neonates who developed pulmonary hypertension following diazoxide therapy.

ID Code	Sex	Gesta-tional Age at Birth (weeks)	Birth Weight	IUGR	Age at diazoxide initiation	Starting dose (mg/kg/d)	Max dose (mg/kg/d)	Prior history of pulmonary hypertension	Respiratory Support prior to diazoxide therapy	Echocardiogram prior to diazoxide therapy.	Echocardiogram on diazoxide (+days on diazoxide)	Respiratory support on diazoxide therapy	Other complications.	Outcome
**G**	M	27 0/7	475g	Yes, with AEDF	11 weeks	10	10	Yes	CPAP 7 cm H_2O, 27% Oxygen	PDA s/p transcatheter closure, IV* septum mildly flattened throughout cardiac cycle.	(+7 days) IV septum moderately flattened throughout cardiac cycle.<br>(+14 days)<br>RV systolic pressure at least 90mmHg (while patient agitated) and 78mmHg (calm) plus RA** pressure. IV septum moderately flattened throughout cardiac cycle.	Conventional ventilator with inhaled nitric oxide	Dubin-Johnson syndrome	Recovered after 4 weeks on ventilator and 7 weeks on inhaled nitric oxide, discharged at 52 weeks corrected gestational age
**H**	F	29 3/7	900g	Yes, with AEDF	6 weeks	5	6.5	No	Room Air	IV septum mildly flattened throughout cardiac cycle	(+13 days) RV systolic pressure at least 62.4mmHg plus RA pressure, IV septum moderately flattened throughout cardiac cycle. Moderate dilation and mild hypertrophy of RV.<br>(+27 days) RV systolic pressure at least 77.8mmHg plus RA pressure. IV septum severely flattened throughout cardiac cycle. Moderate dilation and mild hypertrophy of RV.	High Flow Nasal Cannula, inhaled nitric oxide	Twin, Laryngomalacia requiring supraglottoplasty.	Recovered and returned to room air 10 weeks after diazoxide. Discharged at 48 weeks corrected gestational age.
**I**	M	34 2/7	1320g	Yes	12 days	10	10	No	CPAP 5 cm H_2O, 21% Oxygen	IV septum mildly flattened throughout cardiac cycle, no evidence of PDA	(+7 days) Moderate PDA with bidirectional shunt. IV septum moderately flattened throughout cardiac cycle.<br>(+20 days) Moderate PDA predominantly right to left shunt. IV septum moderately	Intubation and high frequency oscillatory ventilation, inhaled nitric oxide. Hypoxic	Ambiguous genetalia with XY karyotype	Demise during ECLS, related to pulmonary hypertensive crisis.

											flattened throughout cardiac cycle.	respiratory failure leading to ECMO.		
**R**	M	35 0/7	2585g	Yes	2 days	10	10	Yes	Nasal Cannula, 2L/min, 21% Oxygen	RV systolic pressure at least 44mmHg plus RA pressure	(+ 4 days) RV systolic pressure at least 74mmHg plus RA pressure.	CPAP 6, 28%	Deletion of Recurrent Proximal 1q21.1 Region	Recovered and discharged on room air.

IV, Intraventricular
CPAP, continuous positive airway pressure
PDA, patent ductus arteriosus
RA, Right atrium
RV, Right ventricle
AEDF, absent end-diastolic flow
ECMO, extracorporeal membrane oxygenation.
